# Assessment of the feasibility of Juntos: A support programme for families of children affected by Congenital Zika Syndrome

**DOI:** 10.12688/wellcomeopenres.17419.1

**Published:** 2022-03-04

**Authors:** Antony Duttine, Tracey Smythe, Miriam Ribeiro Calheiros de Sá, Silvia Ferrite, Maria Elizabeth Moreira, Hannah Kuper

**Affiliations:** 1 International Centre for Evidence in Disability, London School of Hygiene & Tropical Medicine, London, WC1E 7HT, UK; 2 Instituto Fernandes Figueira, Rio de Janeiro, RJ, 22250-020, Brazil; 3 Dept of Hearing and Speech Services, Federal University of Bahia, Salvador, Bahia, 40110-902, Brazil

**Keywords:** Zika, disability, microcephaly, early intervention, Congenital Zika Syndrome, family, caregiver, Brazil

## Abstract

**Background: **The 2015-16 Zika epidemic resulted in thousands of children born with congenital Zika syndrome (CZS). In Brazil, gaps in the health system often caused parents to be left with insufficient information and support. Consequently, we developed and piloted Juntos - a participatory support programme which aims to improve knowledge, capacities and build support networks for caregivers of children with CZS.

**Methods: **Six caregiver groups received the programme between August 2017 and June 2018: three in Rio de Janeiro and three in Bahia. We assessed the feasibility of Juntos against six of the eight areas of a feasibility framework described by Bowen *et al.* to consider whether Juntos ‘could work’. These areas were: acceptability, demand, implementation, practicality, adaptation and limited efficacy. We used mixed methods including: 1) baseline and end-line questionnaires completed by all group participants; 2) in-depth interviews with 18 participants, seven facilitators and three key stakeholders; 3) participant focus group discussions after each session; 4) researchers session observation; and 5) recording programme costs.

**Results: **37/48 (77%) enrolled families completed both questionnaires. Acceptability and demand were noted as high, based on participant responses to interview questions, focus group feedback and satisfaction scores. Potential for implementation and practicality were also demonstrated through interviews with facilitators and key stakeholders and analysis of project documents. Two groups included caregivers of children with non-Zika related developmental disabilities, showing potential for adaptability. Self-reported quality of life scores increased in caregivers between baseline and end-line, as did the dimensions of family relationships and daily activities in the Pediatric Quality of Life Inventory (PEDS QL) Family Impact Module, showing limited efficacy.

**Conclusions: **The programme showed feasibility according to Bowen’s framework. However, further research of scale up, particularly in the areas of integration, expansion and limited efficacy are needed to ascertain if the programme is effective.

## Introduction

Brazil was the most heavily impacted country in the 2015–16 Zika outbreak, which triggered the World Health Organization to announce a Public Health Emergency of International Concern (PHEIC) ^1, 2^. The causal link between Zika and birth impairments and developmental delays has now been demonstrated ^3^. Congenital Zika syndrome (CZS) is the collective term used to describe the pattern of structural anomalies and functional impairments seen after Zika infection ^4^. This condition includes features such as microcephaly, which was the most associated sign of neurodevelopmental disability during the Zika outbreak. However, not all children display microcephaly at birth and many children may be presenting with Zika-related neurodevelopmental delays who were not detected at birth ^5, 6^. Common conditions and impairments include physical and motor difficulties, intellectual impairments, vision loss and epilepsy. Between November 2015 and March 2020, 18,828 cases of suspected CZS or other aetiologies have been reported to the Ministry of health in Brazil, with 3,523 confirmed cases of CZS ^7^. The states of Pernambuco and Bahia in the North East of the country and Rio de Janeiro in the South East have seen the most cases ^7^.

Children born with CZS are likely to have long-term impairments and disabilities leading to social and economic impacts on families and caregivers, such as depression, anxiety and stress ^8^. Similar trends have been observed in families raising children with other neurodevelopmental disabilities, such as cerebral palsy (CP) ^9– 13^. Children with long-term neurodevelopmental disabilities are likely to need medical, rehabilitative and social support in order to optimise functioning and participation ^14– 16^. In addition, parents and families themselves may have support needs, including education and psychological support ^17, 18^.

The health response in Brazil was mainly focussed on addressing the clinical needs of the children, including provision of medical and therapy services. However, a needs analysis conducted in April-August 2017 showed that there were gaps in service provision and that the needs of children with CZS and their caregivers were not being met though the Brazilian health system alone ^19^. Several formal support groups had been established, but they did not follow a curriculum of training and support. Informal parent support networks existed, such as WhatsApp groups, which were important for parents. However, they did not provide structured guidance and support. The needs assessment highlighted that provision of a structured support programme was an emerging priority within the context of the Zika outbreak ^19^.

In response to these unmet needs, we adapted an existing programme that had been developed for children with cerebral palsy in low- and middle-income contexts. *Getting to Know Cerebral Palsy* (GTKCP) was developed in South Asia for settings where parents had little or no access to formal health or social services ^20, 21^. The programme involves a series of structured sessions around different aspects of caring for children with CP. It has been implemented in many countries and settings across the world. A later adaptation – the Early Intervention Programme (EIP) - has been developed for families of children under two years ^22^. We hypothesised that adapting GTKCP to the Zika context in Brazil would be a feasible and efficient approach, given the likely similarities in experiences of caregivers of children with CP to those with CZS ^19^.

### The intervention

Juntos (meaning ‘together’) was developed from GTKCP and EIP using an evidence-based approach ^23^. The programme aims to improve knowledge, capacities and build networks of support for caregivers of children with CZS. Each week focusses on a different topic and covers the basics of child development and developmental delays, practical sessions such as facilitating play, feeding and communication, and social sessions such as knowing rights and living in the community. The groups are facilitated by a partnership between a therapist (physiotherapist, occupational therapist or speech and language therapist) and a mother of a child with CZS (“expert mother”). The combination of a therapist/mother was chosen to bring a balance of different expertise to the sessions. Facilitators were trained over a one-week period and received support from supervisors during the programme. A coordinator, at each location, oversees the planning and implementation of the programme including recruitment of facilitators and participants, identification of training locations and coordinating logistics for hosting the sessions. Children attend the sessions but were cared for in another space to allow fuller engagement of the caregivers. A full description of the development of the intervention and the Juntos programme is available in a separate paper ^23^.

The aim of this paper is to describe the feasibility of implementing the pilot Juntos programme in two settings in Brazil.

## Methods

The feasibility study was undertaken in partnership with the Instituto Fernandes Figueira in Rio and the Federal University of Bahia in Salvador, with each site nominating a site coordinator. Ethics approval was obtained in both Brazil (IFF/ FIOCRUZ - RJ/MS 2.183.547) and the UK (LSHTM Ethics number 13608). A protocol for the project, including the feasibility assessment, was established ^24^.

### Intervention implementation

Juntos was piloted across six groups in two geographical locations (Rio de Janeiro and Greater Salvador) between August 2017 and June 2018. Groups of six to 10 caregivers of children with CZS were formed, who met weekly in the local community over a period of 10 weeks ^23^.

***Feasibility assessment*.** The holistic evaluation of the programme’s feasibility was structured based on a model proposed by Bowen *et al.* (2010) ^25^ for assessing the feasibility of public health interventions. Bowen *et al.* proposed eight areas of focus to measure, giving an overall picture of the feasibility of an intervention ( Table 1).

**Table 1.** Eight areas of focus from Bowen *et al.* ^25^.

Area of focus	The feasibility study asks:
**Acceptability**	*“To what extent is a new idea, program, process or measure judged as suitable, satisfying, or attractive to program* *deliverers? To program recipients?”*
**Demand**	*“To what extent is a new idea, program, process, or measure likely to be used (i.e., how much demand is likely to exist?)”*
**Implementation**	*“To what extent can a new idea, program, process, or measure be successfully delivered to intended participants in* *some defined, but not fully controlled, context?”*
**Practicality**	*“To what extent can an idea, program, process, or measure be carried out with intended participants using existing* *means, resources, and circumstances and without outside intervention*? *”*
**Adaptation**	*“To what extent does an existing idea, program, process, or measure perform when changes are made for a new format* *or with a different population?”*
**Integration**	*“To what extent can a new idea, program, process, or measure be integrated within an existing system?”*
**Expansion**	*“To what extent can a previously tested program, process, approach, or system be expanded to provide a new program* *or service?”*
**Limited efficacy**	“ *Does the new idea, program, process, or measure show promise of being successful with the intended population, even* *in a highly controlled setting?”*

Bowen *et al.* also proposed that these eight areas can be measured at three different stages of an intervention ^25^ to answer the following questions:

1. “Can it work?” Is asked at the stage when the intervention is being developed and piloted.
2. “Does it work?” Is asked when positive preliminary results have been shown and the intervention has been formally tested.
3. “Will it work?” Is asked at the stage when an intervention has shown to work and aims to be adapted or scaled up.

While this intervention is derived from an existing intervention, Juntos contained new elements and was being delivered to a pilot group in Brazil for the first time ^26^. Therefore, this research project aimed to primarily answer the question of ‘Can it work?’, but with some attention given to explore ‘Does it work?’ and ‘Will it work?’. For our feasibility assessment, we focused on six of the eight areas of Bowen *et al.*’s framework: acceptability, demand, implementation, practicality, adaptation and limited efficacy. The other two areas (integration and expansion) were difficult to measure reliably given the small scale of the pilot project.

The data for the feasibility assessment was collected between August 2017 and June 2018 within the context of six pilot groups implemented in two locations in Brazil – Rio de Janeiro and Greater Salvador, which were both heavily impacted by the Zika outbreak ^7, 27^. Four researchers were recruited by the site coordinators in collaboration with the London School of Hygiene and Tropical Medicine (LSHTM) team. The researchers’ role was twofold: to observe sessions and conduct rapid feedback with participants and facilitators in order to inform fast-track learning for adjusting and honing the programme; and to facilitate baseline and end-line questionnaires, and participant and facilitator semi-structured interviews. The researchers were all female and all had a background in psychology, though this was not a pre-requirement for the position. A two-day training workshop was held for the researchers before the first group, followed by a one day updating session between the first and second groups of each location.

All participants who took part in the programme completed a consent form, relevant to their involvement in the study (e.g., questionnaires, interview). Participants were also requested to provide consent for photographs or other media to be recorded during the group sessions, after explaining that non-agreement to the media consent form would not impact their position in the groups.

We collected a range of data and utilized various methods to assess the six selected areas of Bowen *et al.* This allowed us to analyse the data and acquire a richer understanding of the different facets of each area:

**Participant data**

Baseline and end-line programme semi-structured quantitative questionnaires were completed by all programme participants before the first session and after the last session. If caregivers from the same family came together (e.g., mother and father, mother and grandmother), they would complete one questionnaire per pair with the primary caregiver (usually the mother) as the lead responder. Questionnaires included the following items ( *Extended data* ^28^):

- Socio-demographic characteristics of the child and caregivers (usually mother and father) (baseline only).
- Understanding and knowledge about the child’s condition by the caregiver.
- Knowledge and confidence to care for child, assessed as a five-point Likert scale.
- Health status of child (including questions on general health, serious health issues, seizures and sleep)
- Eating and drinking status of child (including questions around difficulties feeding, level of support, weight gain etc)
- The PedsQL Family Impact Questionnaire Module (Brazilian Portuguese version) ^29, 30^.
- A Cantril scale for assessing quality of life of caregiver and of child ^31^, implemented as a 10 point ladder.
- Review of goals achieved (end-line only).
- Satisfaction (scored out of five for satisfaction of content, organisation and facilitators) and qualitative reflections on the programme (end-line only).

Questionnaires were developed in English, then translated into Portuguese.

The baseline and end-line programme questionnaires were particularly useful in assessing acceptability, demand and limited efficacy areas of feasibility.

In-depth, semi-structured interviews were undertaken face to face by the researchers with 18 participants (16 female, two male) after each group’s final session. Participants were selected purposively at the researchers’ discretion to reflect a broad a range of perspectives (e.g., caregivers of children with different disability severities, mothers and fathers). Since the researchers had been observing all the sessions, they were all known to the participants before the interview. Interviews were undertaken in Portuguese by the local researchers and focussed on satisfaction with and perceived impact of the groups using an interview guide ( *Extended data* ^28^) developed by the lead author. The guide was not piloted before the first interviews. The interviewers recorded and transcribed the interviews in Portuguese and they generally lasted 30–45 minutes. Participants were usually with their child.

Participant data was used to evaluate the acceptability, demand, implementation and limited efficacy areas of feasibility.

**Facilitator data**

In-depth interviews were undertaken with each of the facilitators (total = 7) at the final session of the final group. These were undertaken face to face in Portuguese by the local researchers, who recorded and transcribed the interviews. The interviews focused on the facilitators’ reflections and lessons learned, including perception of participant engagement and the impact of using an interview guide ( *Extended data* ^28^).

Facilitator interviews were used to evaluate the implementation, practicality and adaptation areas of feasibility.

**Key stakeholder data**

In-depth interviews were conducted with the two site coordinators and a senior medical provider. The interviews focussed on the practical components of implementing the sessions, reflections on lessons learned and potential future expansion using an interview guide ( *Extended data* ^28^). Interviews were undertaken in English by the study lead (AD) and were transcribed but none returned to the participants.

Key stakeholder data was used to evaluate the practicality and adaptation areas of the feasibility.

**Other data**

Session costs were assessed by analysing the budget, establishing an overall cost for delivery of the programme, and the cost per participant. Facilitator training costs were calculated and presented separately, as they may not reflect the true costs if the programme was to be scaled up (e.g., the number of facilitators, international travel, etc.). These are variable costs that would need to be estimated at the start of each new programme.

The researchers observed the sessions and used an observation framework to record notes on the: logistics (e.g., timeliness), environment created by facilitators (e.g., room set up), response of caregivers and parents (e.g., contributions of participants) and response of children (e.g., volunteer caregivers’ presence)

Costs and researcher observations were used to evaluate the implementation and practicality areas of feasibility.

**Data analysis**

Analysis of the interviews and session notes/focus groups was undertaken by a social scientist fluent in English and Portuguese who coded the interview responses in NVIVO 12 (Taguette is an alternative open-sourced software). Thematic analysis was structured in advance and centred around the six areas of feasibility being used for this assessment ^25^. The lead author then reviewed all interviews in English and the Portuguese translated elements.

Quantitative data on participant demographics, PEDSQL Family Impact Questionnaire, Cantril Scale and satisfaction were tabulated into Microsoft Excel. Two tail T-testing was performed for the PEDSQL and Cantril Scale. Only questionnaires which had both base- and end-line questionnaires completed (n=37) were used for analysis of PEDSQL and Cantril Scale. All baseline questionnaires (n=48) were used for analysis of the participant demographics.

## Results

A total of 48 families enrolled in the Juntos programme across the six groups (Salvador n=25, Rio n=23) and completed a baseline assessment. 37 families (77%) completed the programme and undertook the end-line assessment, with a slightly higher completion rate in Salvador (84%) than Rio (70%). The number of family units enrolled in each group ranged from seven to 10 (average = eight). The average age of the children of the caregivers at the time of the first session was 23 months (range 13–58 months ^1^). Table 2, below, summarizes the six groups.

**Table 2.** groups, dates and participant numbers.

Group	Dates	Number of families who enrolled	Number of families who completed the end-line questionnaire
Salvador 1	11 ^th^ August – 17 ^th^ November 2017	8	6 (75%)
Salvador 2	20 ^th^ January – 19 ^th^ May 2018	10	8 (80%)
Salvador 3	13 ^th^ March - 13 ^th^ June 2018	7	7 (100%)
Rio 1	17 ^th^ August – 21 ^st^ November 2017	7	7 (100%)
Rio 2	11 ^th^ January – 26 ^th^ April 2018	7	3 (43%)
Rio 3	26 ^th^ February – 6 ^th^ June 2018	9	6 (67%)
		**48**	**37 (77%)**

### Caregiver demographics

The mother was the stated primary caregiver in 46/48 (96%) of the baseline assessments. Most mothers (75%) self-reported that they were married, with 6% divorced and 19% single. A 69% portion of the fathers were living with the mother and 83% of the fathers saw their child on a daily basis. Only three fathers (6%) reported that they had not seen their child in the past six months. The ages of the mothers and fathers are shown in Table 3 below:

**Table 3.** Ages of mothers and fathers.

Age (years)	Mother (n=48)	Father (n=37) ^2^
15–20	5 (10%)	3 (8%)
21–25	17 (35%)	8 (22%)
26–30	5 (10%)	8 (22%)
30–40	18 (38%)	12 (32%)
40–50	3 (6%)	6 (16%)

Only 13% of mothers reported being employed compared to 70% of fathers. 92% of the mothers said they were not working because they needed to care for their child.

The participant, facilitator, key stakeholder and other data that was collected are grouped below following the six selected areas of Bowen *et al.*’s framework: acceptability, demand, implementation, practicality, adaptation and limited efficacy.

### Acceptability

Satisfaction with the programme, as assessed in the end-line questionnaires, was scored highly by the participants (n=35), with an average score of 4.6 (out of 5) for content, 4.8 for organization and 4.7 for facilitators. Group attendance had an average of 6.3 participants per session (based on data from four out of the six groups) ranging from 1 to 17.

During the interviews, a number of caregivers spoke about how being able to engage and share with other caregivers in similar situations was an attraction:

*“I could notice that there were mums in the same situation as me, mums with bigger weaknesses than me, others stronger than me, so I saw it all, and this programme was very important.”* (Participant, Rio)
*“I think that the main thing - the most important thing - is the sharing of experiences, because although there is microcephaly, every case is different, right? So, my daughter has her characteristics, the daughter of another has other characteristics, but I think that sharing experiences is a big plus, right? "Look, I did this, and it worked out, I do it this way". The sharing of experiences is very valid.* (Participant, Rio)

There also appeared to be recognition that the programme focused on areas that the caregivers felt they needed guidance and support with and provided psychological comfort:

*“The programme is interesting…the purpose of this programme is to make the mother be able to administer the issue of microcephaly in a good way...it's not easy, not only for the parents, but for the family, and daily life is complicated...the logistics of everything. There's also the emotional side. So, I think the idea of this programme contributing to helping administer things better is great.”* (Participant, Rio)
*“I think it supported us a lot, totally supported us. It’s like the group was a huge hug. It was a hug, it was what we needed, the support of someone, greater than even ourselves. So I guess support was everything. It changed everything for us. It pulled us up again because everyone was already down. It helped us a lot.” (*Participant, Salvador)

Having a mother expert as a facilitator was also deemed as important/advantageous by other caregivers, as well as by the therapist facilitators:

*“I think the dynamics are great! Very good! I think it's essential that you have a mother, or an aunt, or a grandmother, that a caregiver is one of the facilitators.”* (Therapist facilitator)

However, it was more challenging to get fathers to commit and stay for all the sessions of the programme. When fathers did come, they would not always come to every session or be able to stay for the whole session. Part of this low attendance was related to sessions being offered during the week, when many fathers were working. Furthermore, one father who did attend, and was interviewed, suggested that the format may not be the most natural environment for fathers, who he felt tend to be more timid in interacting in group settings.

### Demand

Out of the forty-eight families who were enrolled in the groups, thirty-seven (77%) attended the final session and provided end-line questionnaires (Salvador n=21, Rio n=16), Table 2. In Salvador, fifteen (60%) families attended at least seven sessions, but only four (16%) came to all 10 sessions. Reasons for dropping out or non-attendance included sickness or death of the child, difficulties with work schedules and transportation, and having too many other appointments and commitments. Recruiting families was a challenge for two of the groups and one, in Greater Salvador, needed to restart due to low numbers.

Despite these challenges, the interviews revealed that the Juntos programme potentially filled an important gap that was not currently being provided though the formal health system or other services. This was reflected by participants, facilitators and key stakeholders:

*“And I still hadn't had personal contact with mothers in the same situation as me, I had seen them on TV or here in the corridors, but I didn't have the opportunity to sit and talk, "How is your life, is it similar to mine?"* (Participant, Rio)

*“There were [other] mothers who wanted to participate, but they couldn't and wanted to join on the fourth or fifth module, and we said they couldn't, but that when a new one opened we would let them know.”* (Parent facilitator, Rio)

### Implementation

The six-pilot group programmes were successfully completed in two major cities of Brazil, suggesting that the programme can be effectively delivered within urban areas. The direct observation revealed that sessions flowed well and that there were good levels of interaction. Facilitators reported that the structure of the sessions and the facilitator guide made the programme logical and easy to lead:

*I think that 'Our Child’* [session 2] *went well and easy to lead, the ‘Play and Early Stimulation' as well [...] and diet, was easy, and the module I think that worked the best, and answered most doubts for everyone was 'Daily Activities’. Nobody included their children in activities, apparently, and there, well, it opened a horizon of options for them”* (Facilitator)

A second room was used as a creche/play area for the children of the caregivers, which allowed the caregivers to be fully engaged in the process. A team of volunteers looked after the children during the sessions. This was positively reflected in the facilitators’ interviews:

*The fact that there were two rooms, I think that it was a positive point, because it's a moment that … not that they stop being mothers, but they leave aside a bit the "I have to live for my child, everything is for my child" and look at themselves as people.* (Facilitator)

### Practicality

The site coordinators spent time in identifying a suitable community location that was accessible for participants. These varied from primary health facilities, from the office of an NGO to a school. This consideration was appreciated and deemed as important:

*“I liked it, I think it was an easy place to get to, near metros, trains, you know?”* (Participant, Rio)

During their interviews, the coordinators also highlighted key features that they identified for a space to make the group sessions work: sufficient space to arrange participants in a circle, space to have the children close to parents (ideally in another room, close by), in a safe location, on the ground floor (or elevator available), accessible by public transportation, and no more than a 30-minute travel away.

Observation notes do suggest that at times the logistics could be challenging, with sessions often starting behind schedule and some not being able to finish all of the content on the same day. The time management aspect did seem to improve in later groups as facilitators became more confident and familiar with the content.

We calculated the average cost to run a 10-session group to be 37,300 Brazilian Reais (7,460 GBP ^3^). This includes the salaries of two facilitators for four months, a coordinator for five months and all the group activities (materials, refreshments and transportation). With an average of eight families per session, this amounts to 4,662 Brazilian Reais (932.50 GBP) per family for the entire 10 sessions. It is important to note that the facilities we used for the groups were all made available at no cost, but this may be an added expense to consider if the programme scales up. This cost does not include the cost to run a facilitator training session and assumes facilitators are trained.

### Adaptation

Adaptations were made to the structure of the programme from the first two groups to the last four, as fast track learning and adjustments were made. In terms of location, different settings were used. In Salvador, all the settings were local primary health facilities, but in different parts of the city. In Rio, two sessions occurred at the same location, a central office space used by a local NGO. The other session took place in a school.

The fast-track learning approach, tailoring content as feedback was received, showed that adaptation was able to happen in ‘real time.’ Coordinators and facilitators conducted rapid analysis of feedback and discussion results, which allowed them to make micro adjustments and improve the structure and flow of the sessions. For example, in one of the Salvador groups, the facilitators identified an issue ahead of the session that discussed toileting (none of the children in the group were yet able to use a toilet). They quickly adapted the session so that they could spend more time on practical aspects for that group (e.g., diaper changing).

The final groups in Rio and Salvador both included a number of children with CP. The programme showed that it was adaptable for caregivers of children with Zika and non-Zika-related neurodevelopmental disabilities.

### Limited efficacy

The study was not powered to show impact. Efficacy limitations were assessed in order to identify domains of potential impact for future studies.

Data from the PEDSQL showed improvement in parent-reported outcomes from baseline to end-line across all dimensions except cognitive functioning (see Table 4 below). Baseline scores of the Juntos participants were similar to those recorded using the same scale in another study which focused on the social and economic impacts for caregivers of children with CZS ^8^. Only two of the PEDSQL were statistically significant: Daily activities (p=<0.001) and the Family Functioning Summary Score (<0.001).

**Table 4.** Changes from baseline to end-line scores across the dimensions of the PEDS QL.

Dimensions of PedsQL	Baseline (n=36) mean (SD)	End-line (n=36)	p-value (t-test)
Physical functioning	50.1 (37.5)	51.8 (37.2)	0.46
Emotional functioning	57.3 (35.1)	60.2 (33.4)	0.29
Social functioning	59.1 (39.9)	59.3 (38.2)	0.95
Cognitive functioning	66.5 (34.1)	61.7 (32.7)	0.06
Communication	60.2 (41.4)	64.1 (36.9)	0.40
Worry	36.1 (41.7)	38.0 (42.8)	0.50
Daily activities	33.5 (36.8)	48.3 (38.5)	<0.001*
Family relationships	60.6 (37.3)	66.4 (32.8)	0.07
**Total score** **Parent HRQL summary** **Family functioning summary**	**53.4 (16.9)** **57.8 (37.0)** **50.6 (39.3)**	**56.0 (14.0)** **57.9 (35.6)** **59.7 (36.0)**	**0.36** **0.96** <0.001*

In the Cantril Scale measurement, there was an increase from 5.6 to 6.5 (out of 10) for the self-reflection on happiness of the caregivers between baseline and end-line (n=37, p=0.007). There was no change in the perceived happiness of the child (n=35, 7.3 at baseline, 7.4 at end-line, p=0.48).

Qualitatively, there were several positive reflections which emerged from the interviews relating to different focus areas of the programme. One mother, for example explained how Juntos helped her with feeding and caring for her child outside of the home:

*“Today she eats very well. The programme helped me a lot with that. When I arrived, I was lost on how to deal with [child] outside the house, at home I had her under control, completely, but out, how would it be?”( Participant*, *Rio*)

Another potential impact is the increased confidence and trust for mothers to be able to leave their children for periods of time with relatives or even in daycare. One mother explained how seeing her child doing so well with the volunteers has given her confidence to pursue job opportunities:

*“I already have a clearer idea of the fact that I want to put my daughter in daycare if I need to, especially if I get accepted into the job I applied to. Before I would say, "I don't want to take the job because I don't have the courage to leave [child] but now, no, I will already look for a place because I saw that it is a good thing”* (Participant, Rio)

Another mother described how being able to leave her daughter with someone else was a goal that she had achieved:

*“[The goal] was to be able to spend a little time without her, because we are always together, yes...I think it was good for her as well as for me, for me it was great. Because we need to have these moments, you know? Because it's me and her the whole day, -twenty-four hours a day, like glue, I think it was good for her because she learnt to be a bit less attached to me and learnt that other people can also treat her the same way, to caress her, and it doesn't need to be me.”* (Participant, Rio)

A third mother shared how the programme had allowed her to take the pressure off herself and accept others to provide support.

*I learnt that we can count on other people, that it doesn't mean that it can only be the dad or the mum, or her little brother, but that we can look for support in other family members like her uncle, godmother, godfather. That we need to trust to leave her with these people. So it was great that I learnt that here in the group too. That we have family members, so why don't we seek their help? Not only these family members but the community as a whole.”* (Participant, Salvador)

## Discussion

The study generated evidence supporting the feasibility of the Juntos programme in several core domains. Our findings have similarities to studies on the Getting to Know Cerebral Palsy programme in Ghana ^21^ and on the Early Intervention Programme in Uganda ^32^.

Juntos has demonstrated acceptability. Participants valued being given an opportunity and an outlet to process their emotions and share day-to-day life experiences in a safe place with their peers. The potential for demand was also demonstrated. Participants, facilitators and key stakeholder interviews all suggested that parents of children with CZS and other neurodevelopmental disabilities would likely welcome the programme across Brazil. This echoed the key findings from the needs analysis that was conducted ahead of the study ^19^. However, recruitment of participants was somewhat of a challenge and the completion rates and attendance of some sessions could have ideally been higher. Further exploration is needed to understand why some participants discontinued the programme. Implementation and practicality were demonstrated to a certain extent. The combination of a mother expert and therapist facilitator seems to work well, and the two differing profiles brought a complementarity to the approach. The Juntos manual was reported to be easy to follow by the facilitators and sessions took place in a range of settings. Location was an important factor for the participants and the provision of transport and refreshment options, although adding to cost, seems to have been another positive factor for the group participation. As a pilot initiative, costs at 7,460 GBP per group were relatively high, but we believe they could be potentially reduced if implemented at scale. A level of adaptability has been demonstrated by implementing the programme in two different contexts and in different settings, and the ability of groups to encompass children with non-Zika-related neurodevelopmental disabilities.

The suggestion of positive caregiver outcomes in the PEDSQL, in the dimensions of daily activities and in the Family Functioning Summary Score, is encouraging. It is logical to observe improvements in these areas, given the nature of the programme and its focus on strengthening the networks providing support to the immediate caregivers. Improvements in the Cantril Scale for perception of quality of life among caregivers also points to potentially promising efficacy of the programme. Some of these sentiments were echoed in the participant interviews, such as the mother who had begun to recognise that raising her child was everyone’s responsibility in the family. The reflections from some caregivers on increased confidence to leave their child with others is encouraging. Some mentioned that this may allow them space to look for work opportunities, and given that only 13% of mothers worked (compared to 70% of fathers) this demonstrates potential impact for the mother and the family’s economic security. 

It is important to note that the sample numbers were too small to make any broad conclusions/generalizations on efficacy limitations. Given that the focus of the intervention was on the caregiver rather than directly on the children, and the relatively short (10 week) duration, we did not hypothesize that there would be any significant change in the functional status of the child and this was shown by our findings. The reasons for the decline in the cognitive functioning dimension of the PEDSQL, which includes aspects such as attention, remembering and thinking quickly, are not clear. As with the positive results, few conclusions can be drawn with such a limited sample size. However, if Juntos is scaled up, further scrutiny may be warranted. Baselines of the PEDSQL scores were broadly similar to those taken from a different cohort of caregivers for a social and economic impact study ^19^.

Scaling up the Juntos programme to other areas of Brazil will take important considerations. However, there is potentially a strong case for doing so: a high need has been demonstrated; it can be extended for families of children with other neurodevelopmental disabilities; it fills an existing gap at community level for families and, with a wide network of community services through healthcare or other sectors, there is potential for its integration into existing structures. However, this will require investment and it would be pertinent to continue to research any future programmes, particularly looking at the areas of integration, expansion and limited efficacy. 

### Strengths and limitations

The study had a number of strengths and limitations, which need to be taken into account when considering the results. Strengths included the use of a mixed methods approach (e.g., questionnaires, interviews, focus groups, data analysis) to assess the six areas of feasibility. This allowed, to a certain extent, for triangulation of these different sources of data. For example, acceptability from participants could be assessed through satisfaction scores in the questionnaires, through interviews and focus groups. We collected data from two different settings and across six groups, which allowed for comparisons between groups and locations. Having two researchers per group was also a strength. Observations could be validated, and different viewpoints reflected.

In terms of limitations, the small sample size means it is not possible to draw any firm conclusions with regards to limited efficacy of the programme, despite the promising results seen in the PEDSQL and Cantril ladder. Furthermore, the selection of interview participants, although done to gather a range of perspectives, may have introduced an inherent bias to the qualitative data that was analysed from the participants. Reasons for participants dropping out of the group were not fully explored in this pilot test, which limits any conclusions made on acceptability, demand and/or practicality. Finally, since the focus of the research was on people already in the programme, we were unable to ascertain reasons why those who never enrolled, chose not to partake in Juntos.

## Conclusions

Juntos has shown that it potentially ‘can work’ according to six of Bowen *et al.*’s eight areas of feasibility. Nevertheless, more research is needed before conclusions can be drawn about whether it ‘will’ or it ‘does’ work. This will require scale-up of the programme to capture data from a wider number of participants. A scaled-up programme would also allow measurement of integration and expansion, which were two areas of feasibility that this pilot did not explore.

## Data availability

### Underlying data

Data associated with this study will not be made freely available, owing to the small number of children with CZS, making data potentially identifiable, and the sensitive nature of the subjects discussed in the interviews and from the questionnaires. However, we are committed to collaborating with other researchers in the analysis of our data (full questionnaire available online). Applications for access to the raw data for this study should be made by contacting Professor Hannah Kuper ( hannah.kuper@lshtm.ac.uk), or Mr Antony Duttine ( antony.duttine@lshtm.ac.uk) and outlining the purpose of the proposed analyses and the variables requested. These applications will be reviewed by the three researchers, and if accepted, the requested variables will be shared.

### Extended data

Open Science Framework: Assessment of the feasibility of Juntos: A support programme for families of children affected by Congenital Zika Syndrome, https://doi.org/10.17605/OSF.IO/AFYBS ^28^

This project contains the following extended data:

- 3. pre and post questionnaires.xlsx
- Qualitative interview questions - participants.docx
- Qualitative interview questions - facilitators.docx
- Qualitative interview questions - key informants.docx

Data are available under the terms of the Creative Commons Attribution 4.0 International license (CC-BY 4.0).

## Footnotes

^1^ The 58-month-old was a non-Zika child.

^2^ Although we collected information on 37 fathers, not all of them participated in the sessions. The mother/primary caregiver was asked to provide information about the father even if they did not attend.

^3^ GBP/BRL exchange rate of 4.96 taken from xe.com for the 13 ^th^ June 2018 (last day of the last Juntos session)
