# Supplementary material for: Assessment of the feasibility of Juntos: A support programme for families of children affected by Congenital Zika Syndrome
Source: Wellcome Open Res. 2022 Mar 4;7:77. [Version 1] doi: 10.12688/wellcomeopenres.17419.1 (PMC9127372; doi:10.12688/wellcomeopenres.17419.1)
Supplement: Peer review history

# Peer review: Reviewer response for version 1

**DOI:** 10.21956/wellcomeopenres.19258.r49915

**Published:** 2022-05-23

**Authors:** Sarah Mulkey

**Affiliations:** 1 Prenatal Pediatrics Institute, Children’s National Hospital, Washington, DC, USA; 2 George Washington University School of Medicine, Washington, DC, USA

Thank you for the opportunity to review this manuscript. It was well written and described a special support program for families of children with congenital Zika syndrome. The quotes of the participants show just how important the program was for them and it sounds that it has made a difference for the families that participated. Families often benefit so much by the support of other families going through similar situations. The program seems well structured and that it could be refined to enhance program completion and session timelines. A follow-up of how lasting the benefits of the program are for the families would be very interesting.

*"Are all the source data underlying the results available to ensure full reproducibility?" *- Partly, but the authors acknowledge this and provide a valid reason of confidentiality, given the small sample size. Full data can be requested, and considered upon review to be shared.

 Some additional questions:

1. Can the authors please provide more detail of the PEDS QL and how this is scored. Is the total score a sum of all of the dimensions? What values are considered high and low?
2. I really loved the direct quotes! They were beautiful and very descriptive about the experiences of these families.
3. Why do the authors think that the Rio 2 cohort had the lowest number of families who completed the program? What was different about this particular group?
4. These cohorts met prior to the COVID-19 pandemic. Can the authors provide any information about any support that has continued or how support has been disrupted for these families?
5. Can the authors discuss any changes that they feel would benefit from the program based on what they learned regarding the feasibility?

Is the work clearly and accurately presented and does it cite the current literature?

Yes

If applicable, is the statistical analysis and its interpretation appropriate?

Yes

Are all the source data underlying the results available to ensure full reproducibility?

Partly

Is the study design appropriate and is the work technically sound?

Yes

Are the conclusions drawn adequately supported by the results?

Yes

Are sufficient details of methods and analysis provided to allow replication by others?

Yes

Reviewer Expertise:

Child neurology, neurodevelopment, congenital infections

I confirm that I have read this submission and believe that I have an appropriate level of expertise to confirm that it is of an acceptable scientific standard.

# Peer review: Reviewer response for version 1

**DOI:** 10.21956/wellcomeopenres.19258.r49027

**Published:** 2022-04-14

**Authors:** Paul Lynch

**Affiliations:** 1 Inclusive Education, School of Education, University of Glasgow, Glasgow, UK

Juntos programme is aimed at caregivers whose children have Congenital Zika Syndrome and other neuro-developmental disabilities in Brazil. The 10 session programme covers facilitating play, feeding and communication, knowing rights and living in the community. The paper is clearly set out, very accessible to read and refers to up-to-date literature. The mixed method design is appropriate to the type of intervention. The Bowen et al. feasibility framework is appropriate to measure the feasibility of the training programme. The data analysis is clear and succinct. The qualitative data provides useful contextual detail about the impact of the sessions on individual caregivers. All the data sources are made clear for reproducibility. 

 It would be helpful to know a little more about the intervention in terms of duration, length of each session and what materials were used to help facilitate the sessions. It's curious why cognitive functioning score was lower after the training. Is there a plausible reason why this was the case?

 The conclusions drawn from the data are concise and not inflated, considering the small number of participants. There is reasonable amount of evidence to show that the intervention has been successful and 'can work'. 

 An important contribution to an under-researched field of disability.

Is the work clearly and accurately presented and does it cite the current literature?

Yes

If applicable, is the statistical analysis and its interpretation appropriate?

Yes

Are all the source data underlying the results available to ensure full reproducibility?

Yes

Is the study design appropriate and is the work technically sound?

Yes

Are the conclusions drawn adequately supported by the results?

Yes

Are sufficient details of methods and analysis provided to allow replication by others?

Yes

Reviewer Expertise:

My expertise lies in early childhood development, education and disability in low and middle income countries. I have a strong background in developing and implementing caregiver and pre-school teacher training programmes for young children with disabilities in LMICs.

I confirm that I have read this submission and believe that I have an appropriate level of expertise to confirm that it is of an acceptable scientific standard.
